# Recent Advances in the Semisynthesis, Modifications and Biological Activities of Ocotillol-Type Triterpenoids

**DOI:** 10.3390/molecules25235562

**Published:** 2020-11-27

**Authors:** Yucheng Cao, Kaiyi Wang, Si Xu, Lingtan Kong, Yi Bi, Xiaopeng Li

**Affiliations:** School of Pharmacy, Key Laboratory of Molecular Pharmacology and Drug Evaluation (Yantai University), Ministry of Education, Collaborative Innovation Center of Advanced Drug Delivery System and Biotech Drugs in Universities of Shandong, Yantai University, Yantai 264005, China; cchm1187829@163.com (Y.C.); wangky_1994@163.com (K.W.); xs14736999@163.com (S.X.); klt13275374569@163.com (L.K.); patricklee@chgb.org.cn (X.L.)

**Keywords:** biological activity, modification, semisynthetic, ocotillol-type triterpenoids

## Abstract

Ginseng is one of the most widely consumed herbs in the world and plays an important role in counteracting fatigue and alleviating stress. The main active substances of ginseng are its ginsenosides. Ocotillol-type triterpenoid is a remarkably effective ginsenoside from Vietnamese ginseng that has received attention because of its potential antibacterial, anticancer and anti-inflammatory properties, among others. The semisynthesis, modification and biological activities of ocotillol-type compounds have been extensively studied in recent years. The aim of this review is to summarize semisynthesis, modification and pharmacological activities of ocotillol-type compounds. The structure–activity relationship studies of these compounds were reported. This summary should prove useful information for drug exploration of ocotillol-type derivatives.

## 1. Introduction

**Figure 1:**
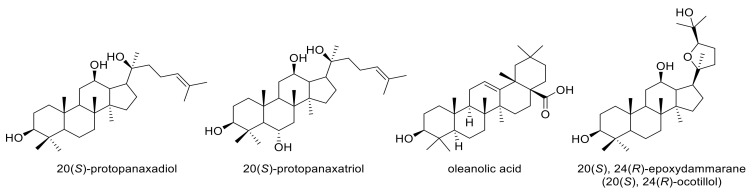
Ginsenosides are found in the highest abundance in Vietnamese ginseng.

From the 1940s to the middle of 2019, approximately 33.5% of approved drugs were either natural products or directly derived from them [1]. The development of new drug entities based on natural products as sources of novel structures is still an area of active research. Ginseng, including Asian ginseng (*Panax vietnamensis* HA et GRUSHV.) and American ginseng (*Panax quinquefolium* L.), is one of the most widely consumed herbs in the world and plays an important role in counteracting fatigue and alleviating stress [2,3]. Ginseng contains a variety of active ingredients, but its main active substances are attributed to its ginsenosides. The ginsenosides with the highest content in Vietnamese ginseng are protopanaxadiol, protopanaxatriol, oleanolic acid and 20,24-epoxydammarane (ocotillol) (Figure 1) [4,5].

Among the active components of ginseng, ocotillol-type compounds have received increasing attention because of their antibacterial, anticancer and anti-inflammatory properties [6,7]. Their different pharmacologic effects and potential molecular mechanisms have been gradually elucidated. Compared with the structure of dammarane ginsenosides (including the protopanaxadiol and protopanaxatriol types), ocotillol-type saponins are tetracyclic triterpenoid saponins containing a furan ring linked with aglycones.

Ocotillol-type saponins were first isolated from *Fozrqwieria splendens* Eliselm. in 1965 by Warnhoff et al. They were also found in *Panax quinquefolium* L, *Panax vietnamensis* HA et GRUSHV, and *Panax japonicus* var, to name a few [8,9,10,11,12,13,14,15,16,17]. However, because of the low content of ocotillol-type saponins in natural products, there were few studies on ocotillol-type derivatives in previous years [18,19]. Fueled by the growing use of semisynthetic methods for the preparation of ocotillol-type derivatives, increased research of ocotillol-type derivatives has been recently observed. In 2016, Liu et al. published a review that focused on the discovery, semisynthesis, biological activities and metabolism of ocotillol-type saponins [6]. However, the structure of most derivatives and its structure–activity relationship (SAR) were not mentioned in the article. Compared with the previous review, this review summarized the semisynthesis, modification and pharmacological activities of ocotillol-type derivatives. All the structures of ocotillol-type derivatives and their SARs in antibacterial, anti-inflammatory and tumor multidrug resistance reversal were summarized. This review provides useful information for the development of ocotillol-type derivatives and gives a direction for further inspiration to enrich its structures with good pharmacological activities.

## 2. Semisynthesis of Ocotillol-Type Compounds

Ocotillol-type sapogenins are less abundant in natural sources. Vietnamese ginseng contains higher amounts of ginseng saponins compared with other *Panax* genus species. The content of ocotillol-type saponins in *Panax Vietnamese* ginsengs is only 5.6%, while in *Panax quinquefolius*, it is less than 0.01% [20]. Additionally, 1 kg of fresh rhizome low-quality Vietnamese ginseng is about $1000 in 2019. These factors may have led to the slow development of ocotillol-type ginsenosides in previous years.

In 2005, 20(*S*)-protopanaxadiol (20(*S*)-PPD) was used as a raw material to obtain **4** and **5** by a semisynthetic method [21]. Yang et al. optimized and improved the synthetic process and achieved the industrial production of **4** and **5** [22].

**Figure 2:**
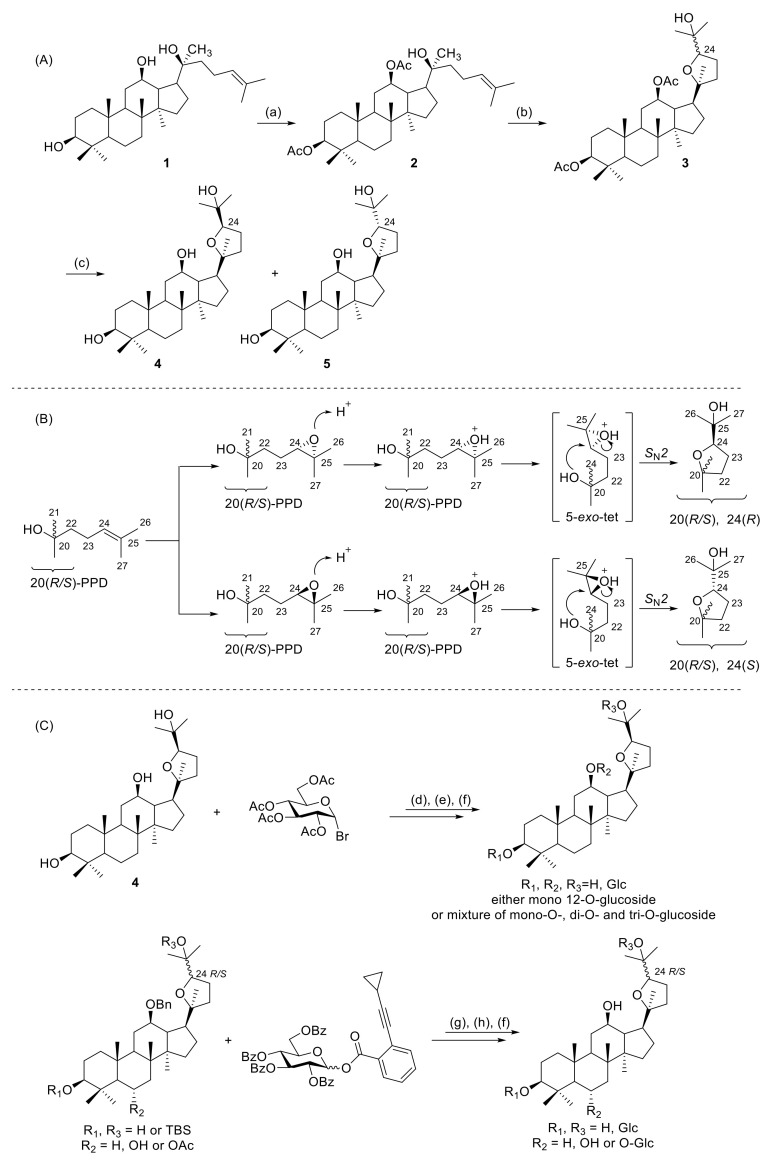
(**A**) Semisynthetic route for the preparation of ocotillol-type epimers. (**B**) Synthetic mechanism of ocotillol-type epimers. (**C**) Synthesis of ocotillol-type ginsenosides. Ac = acetyl; Bn = benzyl; Glc = β-d-glucopyranosyl; Bz = benzoyl. Reagents and conditions: (a) (CH_3_CO)_2_O, DMAP, pyridine, r.t.; (b) *m*-CPBA, CH_2_Cl_2,_ r.t.; (c) NaOH, CH_3_OH, H_2_O, 65 °C; (d) alcohol, mercury cyanide, nitromethane, 90 °C, 1 h; (e) alcohol, *α*-acetobromoglucose, r.t.; (f) KOH/CH_3_OH, THF, r.t.; (g) Ph_3_PAuNTf_2_, CH_2_Cl_2_, r.t.; (h) H_2_, Pd(OH)_2_/C, CH_3_OH, r.t.

Ocotillol-type sapogenins have been made using similar synthetic methods. 20(*S*)-PPD was used as the raw starting material and reacted with acetic anhydride, and then acetylated 20(*S*)-PPD was oxidized by *m*-CPBA. The molar ratio of the acetylated 20(*S*)-PPD to *m*-CPBA at −3 ℃ is approximately 1:4, 3 h. The ocotillol-type epimers (**4**, **5**) were obtained by the hydrolysis of the oxidation products. The synthetic route is shown in Figure 2A [23].

After further research by Meng et al., the synthesis mechanism of ocotillol-type epimers was proposed as follows (Figure 2B). 20(*S*)-PPD or 20(*R*)-PPD is oxidized by *m*-CPBA to generate the 24,25-epoxy intermediates, and then an intramolecular ring-opening loop reaction is carried out according to Baldwin’s rule, and finally cyclization by a 5-exo-tet method forms a tetrahydrofuran ring [24,25,26].

**Figure 3:**
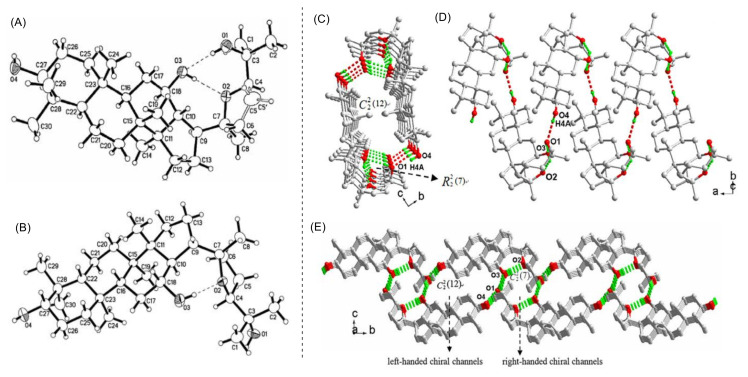
(**A**) The Oak Ridge Thermal Ellipsoid Plot Program for Crystal Structure Illustrations (ORTEP) figure of 20(*S*),24(*R*)-ocotillol-type saponin (**4**). (**B**) The ORTEP figure of 20(*S*),24(*S*)-ocotillol-type saponin (**5**). Thermal ellipsoids shown at 30% probability. (**C**) and (**D**) view of the H-bonded 1D left-handed chiral channel in 20(*S*),24(*R*)-ocotillol-type saponin (**4**). (**E**) The 2D net with right-handed and left-handed chiral channels in 20(*S*),24(*S*)-ocotillol-type saponin (**5**).

Further research proved that the epimerization of C-24 could also result in remarkable differences in both the molecular conformation and the crystal packing arrangements. These remarkable differences may lead to diversity in both polarity and activity of the ocotillol-type epimers. The 24(*S*)-epimer (**5**) had two intramolecular hydrogen bonds, while the 24(*R*)-epimer (**4**) had one intramolecular hydrogen bond (Figure 3A,B) [27,28]. Crystal stacking showed that both the 20(*S*),24(*R*)-ocotillol and 20(*S*),24(*S*)-ocotillol generated an H-bonded tube, the 24(*R*)-epimer (**4**) generated a left-handed chiral channel, while the 24(*S*)-epimer (**5**) extended into the two-dimensional network with right-handed and left-handed chiral channels (Figure 3C–E) [29]. Additionally, the 24(*R*)-epimer (**4**) had weaker molecular polarity compared with the 24(*S*)-epimer (**5**). These differences in hydrogen bonding may contribute to the differences in the observed biological activity and molecular polarity of the 24(*R*)-epimer (**4**) compared with the 24(*S*)-epimer (**5**).

Ocotillol-type ginsenosides are rarely found in nature. Less than 20 naturally occurring ocotillol-type ginsenosides have been characterized and reported [8,9,10,11,12,13,14,15,16,17]. The use of chemical methods to synthesize new ocotillol-type ginsenosides is a promising approach to generate structural diversity. Atopkina et al. reported the synthesis of ocotillol-type ginsenosides by coupling the acceptor **4** with *α*-acetobromoglucose and orthoester donors in the presence of mercury salts (Figure 2C) [30,31]. In 2016, Shen et al. used a gold-catalyzed glycosylation scheme to synthesize ocotillol-type ginsenosides under neutral conditions (Figure 2C) [32]. Many ocotillol-type ginsenosides can be synthesized by this method, and further investigations of ocotillol-type ginsenosides should be pursued.

## 3. Pharmacological Activities and Chemistry

### 3.1. Antibacterial Effects

Evidence has shown that ginseng has antibacterial properties, and its extract may be effective for treating bacterial infections in the future [33]. Compound **5** had strong antibacterial activities against *Staphylococcus aureus* (*S. aureus*) and *Bacillus subtilis* (*B. subtilis*) with minimum inhibitory concentration (MIC) values of 8 μg/mL [34]. Further research showed that **5** also had strong synergistic inhibition against community-associated methicillin-resistant *S. aureus* (MRSA; strain USA300), as **5** reduced the MIC of kanamycin (KAN) against MRSA USA300 from 1 μg/mL to 0.125 μg/mL giving a fractional inhibitory concentration index (FICI) of 0.14.

**Figure 4:**
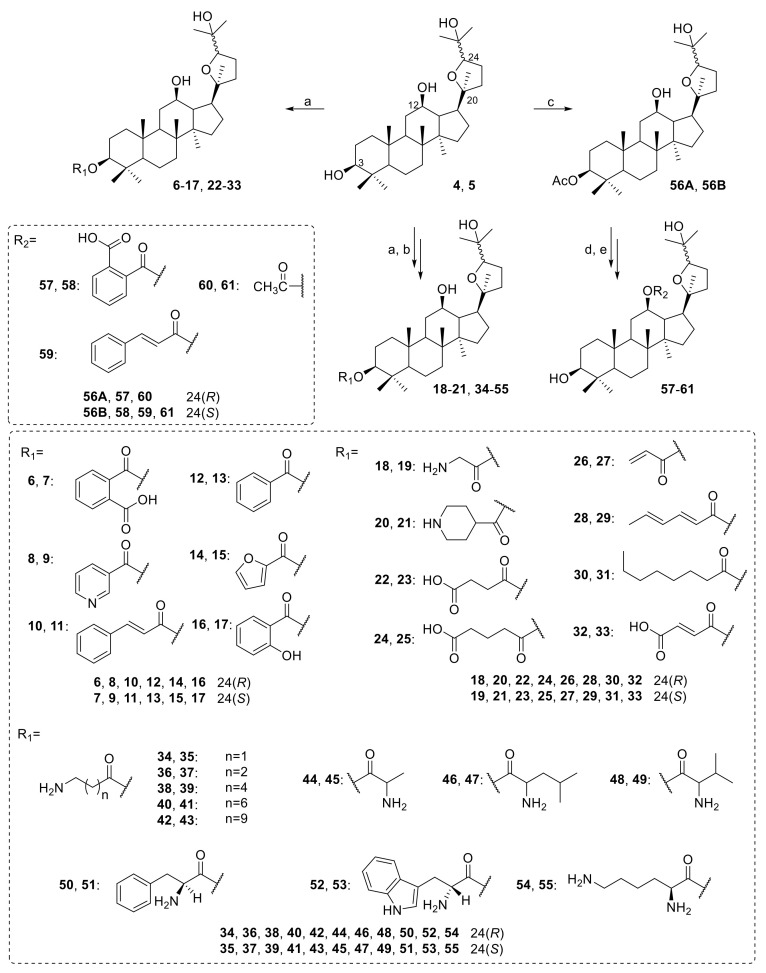
Synthesis of ocotillol-type derivatives 6–61. Reagents and conditions: (a) anhydrous CH_2_Cl_2_, anhydride or acids or Boc-amino acid, 1-ethyl-3(3-dimethylpropylamine) carbodiimide (EDCI), 4-dimethylaminopyridine (DMAP), r.t.; (b) trifluoroacetic acid (TFA), CH_2_Cl_2_, r.t.; (c) anhydrous pyridine, Ac_2_O, DMAP, r.t.; (d) anhydrous pyridine, anhydride or acid chloride, DMAP, ref.; (e) CH_3_OH, KOH, ref.

The furan ring, C-3 and C-12 are possible to explore in terms of chemical diversity as a modification of the furan ring, C-3, and C-12 significantly changed the antibacterial activity of ocotillol-type derivatives. Aromatic-substituted ocotillol-type derivatives **6**–**17** were synthesized by an esterification reaction, and their in vitro activity against *Escherichia coli* (*E. coli*), *B. subtilis*, *S. aureus*, *Pseudomonas aeruginosa* (*P. aeruginosa*) and *Acinetobacter baumannii* (*A. baumannii*) was determined (Figure 4) [35]. Compounds **6** and **7** exhibited excellent antibacterial activities with MIC values of 1 μg/mL against *S. aureus* and *B. subtilis*, while compounds **9**, **10**, **12** and **16** exhibited moderate antibacterial activities against *S. aureus*. Further research showed that **6** and **7** displayed good antibacterial activities against MRSA USA300 with MIC values of 4 μg/mL. Additionally, **6** and **7** combined with KAN and chloramphenicol had strong synergistic inhibition against MRSA USA300 and reduced the MICs of KAN against MRSA USA300 from 1 μg/mL to 0.0156 and 0.0625 μg/mL (FICI = 0.078 and 0.020, respectively).

Bi et al. synthesized aliphatic ocotillol-type derivatives **18**–**33** (Figure 4) [36,37,38]. Compounds **18**, **20**–**23**, **25** and **30** showed good antibacterial activities against *S. aureus* and *B. subtilis*. Further screening results showed that **5**, **18** and **19** had similar antibacterial activities against MRSA USA300 with MIC values of 8 μg/mL. Most ocotillol-type derivatives with an amino group at C-3 displayed excellent antibacterial activities, while those with a carboxylic group at C-3 showed moderate activities. A synergistic effect was observed for compound **19** as it reduced the MIC of KAN against MRSA USA300 from 1 µg/mL to 0.25 µg/mL with a FICI of 0.28.

A series of ocotillol-type derivatives **34**–**55** with an amino group was also synthesized (Figure 4) [39,40]. The antibacterial activity results showed that most of the ocotillol-type derivatives with an amino group had moderate to good inhibitory activities against Gram-positive bacteria but had no effect on Gram-negative bacteria. Compounds **38**, **40** and **51** had good inhibitory activities against MRSA USA 300 with MICs ≤ 4 µg/mL, while **51** had the same antibacterial activity as KAN. A synergistic effect was observed for **39** when it was combined with KAN as shown by the significant enhancement of the MIC from 4 µg/mL to 0.25 µg/mL (FICI < 0.0088) against MRSA USA300.

A series of derivatives **57**–**61** were synthesized and screened. Among them, compound **58** had the best antibacterial activity against MRSA USA300 with a MIC of 8 μg/mL, and **60** had a moderate inhibitory effect against both Gram-positive and Gram-negative bacteria (Figure 4). Additionally, **58** combined with KAN had strong synergistic inhibition against MRSA USA300 with a FICI of 0.008 [34].

The synthetic approaches to prepare compounds **6**–**56B** are only slightly different. Compound **6** was synthesized by the treatment of compound **4**, DMAP and phthalic anhydride in dry dichloromethane over 6 h to obtain **6** with 73% yield at room temperature. 1-ethyl-3(3-dimethylpropylamine) carbodiimide (EDCI) is an excellent dehydrating agent that can accelerate the esterification reaction. Compound **20** was synthesized by the treatment of **4**, DMAP, *N*-Boc-isonipecotic acid and EDCI in dry dichloromethane over 3 h to obtain the intermediate with 80% yield at room temperature. The use of EDCI can increase the speed and yield of the esterification reaction. It is noteworthy that the hydroxyl group at the C-12 does not easily react with anhydride or acid because of steric hindrance and the formation of hydrogens bond. After the addition of **56A**, DMAP and phthalic anhydride to anhydrous pyridine at 120 °C for 25 h, the yield of the intermediate is only 50%.

**Figure 5:**
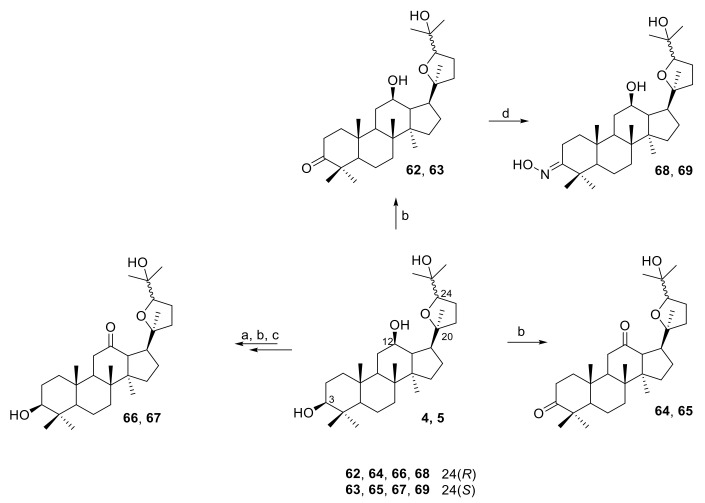
Synthesis of ocotillol-type derivatives **62**–**69**. Reagents and conditions: (a) anhydrous pyridine, Ac_2_O, DMAP, r.t.; (b) anhydrous CH_2_Cl_2_, pyridinium chlorochromate (PCC), r.t.; (c) CH_3_OH, KOH, ref.; (d) anhydrous pyridine, NH_2_OH·HCl, 80 °C.

Ocotillol ketone derivatives **62**–**69** were synthesized by Zhou et al. (Figure 5) [34,36]. Compound **4** (0.21 mmol) in dry dichloromethane (8 mL) was added to pyridinium chlorochromate (0.40 mmol), and the mixture was stirred at room temperature for 3 h to obtain compound **62** with 66% yield. While compound **64** was synthesized by combining **4** (0.33 mmol) and pyridinium chlorochromate (1.00 mmol) in dry dichloromethane (8 mL), the reaction takes about 8 h to obtain intermediate with 76% yield at room temperature. Compound **65** had excellent antibacterial activities against *S. aureus* with a MIC of 16 μg/mL, while compounds **67** and **69** had moderate inhibitory effects against *S. aureus*.

**Figure 6:**
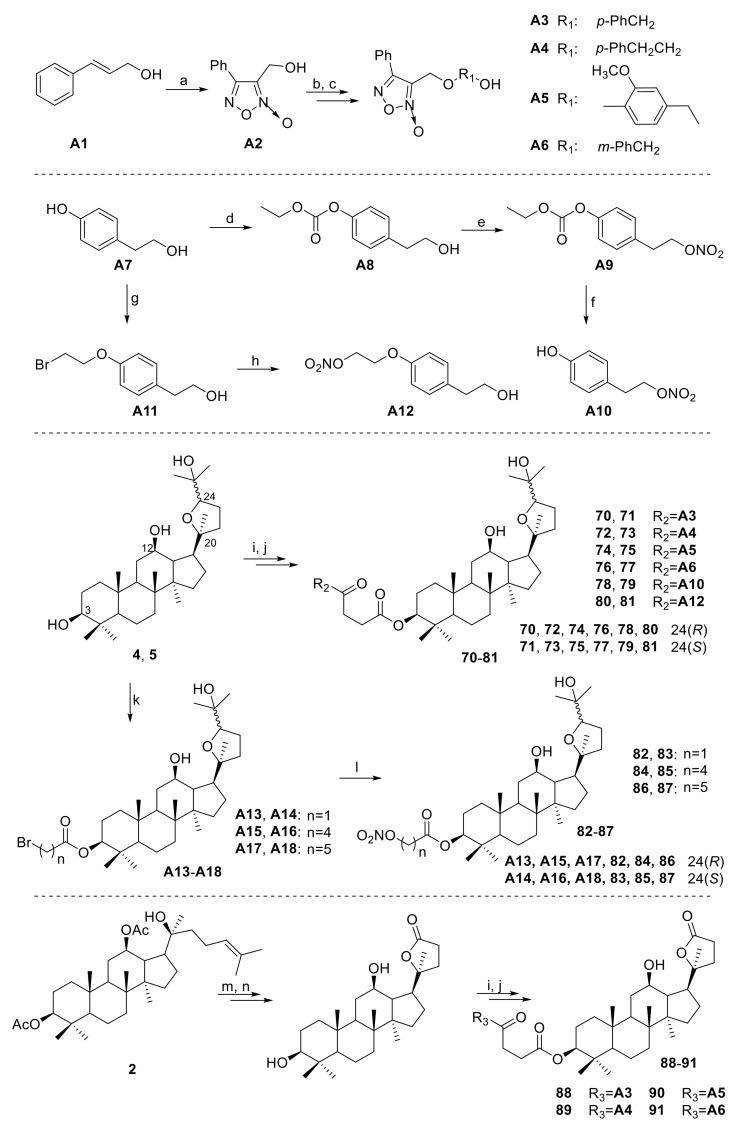
Synthesis of ocotillol-type derivatives **70**–**91**. Reagents and conditions: (a) NaNO_2_, HOAc, r.t., 1 h; (b) SOCl_2_, pyridine, CH_2_Cl_2_, r.t., 8 h; (c) HOR_1_OH, K_2_CO_3_, KI, CH_3_CN, r.t., 3 h; (d) 1 M NaOH, ethyl chlorocarbonate, −5 ℃; (e) Acetic anhydride, nitrosonitric acid, CH_2_CI_2_, 0 ℃; (f) cholamine, ethyl alcohol, r.t.; (g) K_2_CO_3_, 1,2-dibromoethane, THF, r.t.; (h) AgNO_3_, CH_3_CN, 70 ℃, protection from light; (i) succinic anhydride, DMAP, CHCl_3_, 42 °C, 6 h; (j) **A3-6**, **A10**, **A12**, DMAP, EDCI, 25 °C, CH_2_Cl_2_, 6 h; (k) Bromoacetic acid, 5-bromopentanoic acid or 6-bromohexanoic acid, Et_3_N, DMAP, EDCI, dry CHCl_3_, r.t.; (l) AgNO_3_, CH_3_CN, 70 °C, protection from light; (m) CrO_3_, CH_3_COOH, H_2_O, r.t., 3 h; (n) NaOH, H_2_O, CH_3_OH, reflux, 6 h.

Ocotillol-type derivatives with a nitric oxide (NO) donor **70**–**91** were synthesized, their NO release ability and the antibacterial abilities of some derivatives were studied (Figure 6) [4,41]. Compounds **70**–**91** showed similar NO-releasing capability at 100 µM. Compounds **71**, **75**, **77**, **83**, **84**, **86,** and **91** showed better NO-releasing capability at 500 µM as after 30 min of reaction, they all released more than 0.2 M NO. Compounds **83** and **86** demonstrated good activities against Gram-positive bacteria (MIC = 16 μg/mL against *B. subtilis 168* and *S. aureus*). Compound **86** displayed broad-spectrum activity against Gram-positive and Gram-negative bacteria. Compound **86** used with chloramphenicol also showed good synergistic effects with a FICI = 0.03 against MRSA USA300.

**Figure 7:**
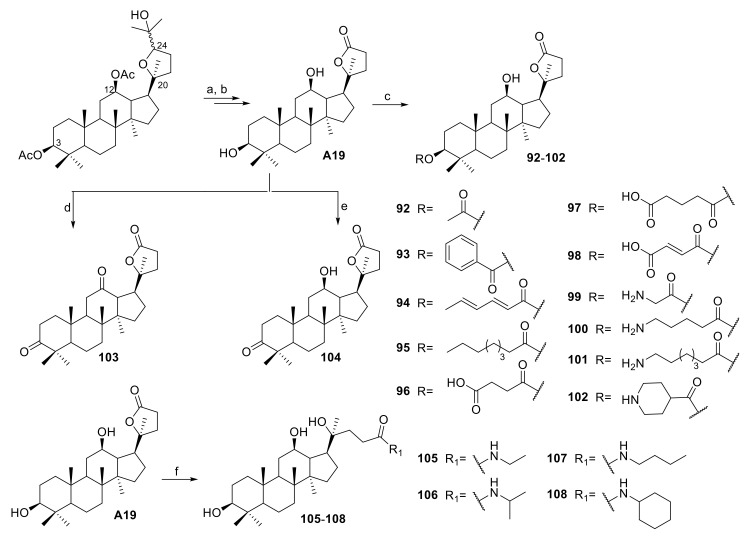
Synthesis of ocotillol-type derivatives **92**–**108**. Reagents and conditions: (a) Jones reagent, acetone, r.t.; (b) (1) KOH, MeOH, H_2_O, 60 °C; (2) 50% H_2_SO_4_; (c) 1) corresponding acid, anhydride or Boc-amino acid, DMAP, EDCI, CH_2_Cl_2_, r.t.; (2) CH_2_Cl_2_, TFA, r.t.; (d) excess of PCC, CH_2_Cl_2_, r.t.; (e) 1 M of PCC, CH_2_Cl_2_, r.t.; (f) NaOH, THF, R_1_NH_2_, r.t.

A series of ocotillol-type lactone derivatives **92**–**108** was designed by Zhang et al. (Figure 7) [42]. Compounds **96**–**98**, **100** and **102** demonstrated good activities against *S. aureus* and *B. subtilis* with MIC values of 1 to 8 μg/mL. Compounds **96**, **100**, **101**, **102**, **105** and **107** showed good activities against MRSA USA 300. Compounds **96** and **102** also exhibited bactericidal activities with minimum bactericidal concentration (MBC) values of 4 and 8 μg/mL. Additionally, **102** reduced the MICs of KAN and chloramphenicol against MRSA USA300 from 1 and 4 μg/mL to 0.125 and 1 μg/mL (FICI = 0.141 and 0.375), respectively. Zhang et al. also analyzed the antibacterial effect of the ocotillol-type lactone derivative **102** by scanning electron microscopy, a cytoplasmic β-galactosidase leakage assay and UV-visible analysis [42]. The results showed that **102** might exert its antibacterial effect by damaging bacterial cell membranes and disrupting the function of DNA. The precise mechanism of its DNA antibacterial action is currently under investigation.

**Figure 8:**
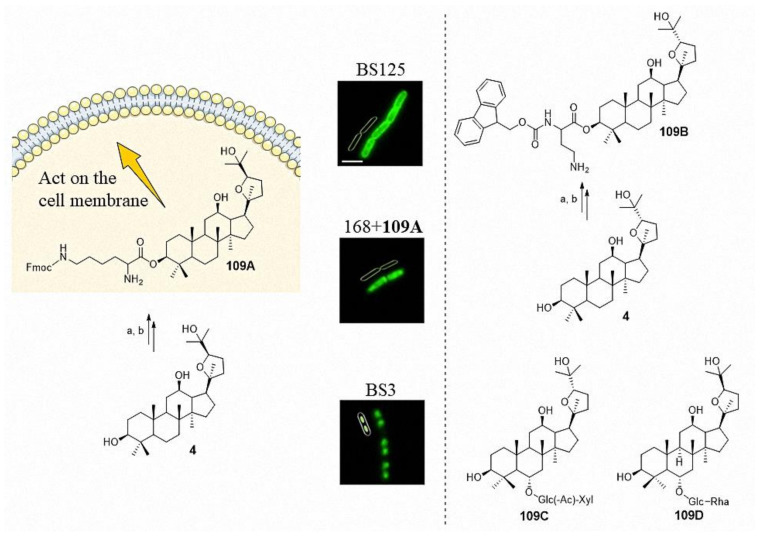
Structure of the ocotillol-type derivative **109A**-**109D** and the epifluorescent microscopy images of *B. subtilis* strain BS125 (top), strain 168 with compound **109A** treatment (middle), and strain BS3 (bottom). Scale bar: 4 μm. Reagents and conditions: (a) DMAP, EDCI, *N*-Boc-*N’*-Fmoc-l-Lysine, CH_2_Cl_2_, r.t.; (b) TFA, CH_2_Cl_2_, r.t.

At present, the antibacterial target of ocotillol-type derivatives is still not clear. Bi et al. synthesized the ocotillol-type probe **109A**, which had a MIC of 8 μg/mL against *B. subtilis*. An epifluorescent microscopy study showed that **109A** was mainly distributed on the bacterial cell membrane rather than within the nucleoid (Figure 8) [4]. On this basis, Bi et al. synthesized the ocotillol-type probe **109B**, which had a MIC of 1 μg/mL against MRSA 18–19 (Hospital-acquired methicillin-resistant *Staphylococcus aureus*, collected in Chengdu, China from 2018) [43]. The antibacterial mechanism of **109B** against MRSA 18–19 is currently underway. The number of ocotillol-type probes is small, which limits the discovery of their antibacterial target. In 2017, 28-hydroxy protopanaxadiol was synthesized as a novel probe template [44]. The synthesis of new ocotillol-type probes employing 28-hydroxy protopanaxadiol may provide an effective means to enrich the structure of ocotillol-type probes. Additionally, functional probes that target the cell membrane are needed. Further research of ocotillol-type probes will promote the discovery of the target protein and provide a reference for the development of more effective drugs.

**Figure 9:**
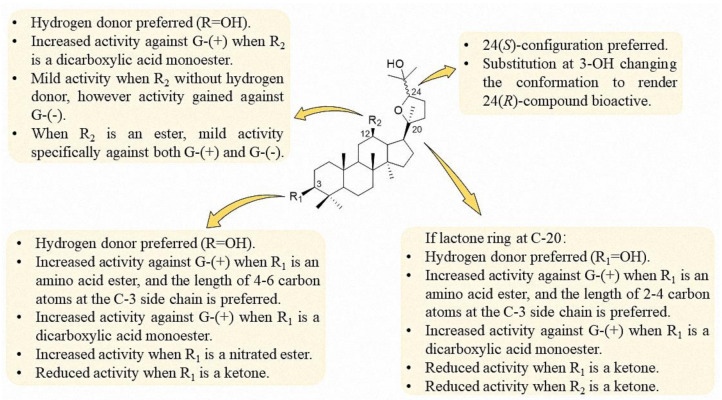
Structure–activity relationship (SAR) of the antibacterial activity of ocotillol-type derivatives.

Based on the present research of the ocotillol-type derivatives, a preliminary SAR of their antibacterial activities is summarized in Figure 9. The 24(*S*)-configuration is preferred, while substitution at the 3-OH changes the conformation to render the 24(*R*)-compound bioactive. A hydrogen donor at C-3 and C-12 are preferred to maintain the activity against Gram-positive bacteria. Decreased activity was observed when the functional groups at C-3 and C-12 were a ketone. When R_2_ is an ester, mild activity against Gram-negative bacteria was observed.

### 3.2. Anti-Inflammatory Activities of Ocotillol-Type Derivatives

Lipopolysaccharide-stimulated RAW 264.7 cells can release the inflammatory mediator NO, prostaglandin E_2_ (PGE_2_), tumor necrosis factor (TNF-*α*), interleukin-6 (IL-6) and anti-inflammatory mediator interleukin-10 (IL-10). The anti-inflammatory activity of 20(*S*)-ocotillol (**4**, **5**) and 20(*R*)-ocotillol (**150**, **151**) was evaluated in RAW 264.7 cells. The results showed that both 20(*S*)-ocotillol and 20(*R*)-ocotillol inhibited the release of the inflammatory cytokines NO and interleukin-6 (IL-6). However, the 20(*S*)-epimers mainly inhibited the release of PGE_2_ and primarily increased the release of the anti-inflammatory mediator IL-10. The 20(*R*)-epimers inhibited the release of the inflammatory cytokine TNF-α [45,46]. Oral ocotillol-type ginsenosides such as **109C** (Figure 8) are metabolized to ocotillol-type sapogenin in the gut by gut microbiota [47]. Ocotillol-type sapogenin showed the highest inhibitory effect. In vitro studies demonstrated that 20(*R*),24(*R*)-ocotillol might ameliorate colitis by inhibiting the expression of the proinflammatory cytokines TNF-α, interleukin-1β, IL-6, interleukin-17 (IL-17), interleukin-23 (IL-23) and interferon-γ (IFN-γ). Additionally, 20(*R*),24(*R*)-ocotillol strongly ameliorated Trinitro-benzene-sulfonic acid-induced iNOS and cyclooxygenase-2 (COX-2) expression, as well as activation of their transcription factors NF-κB and MAPKs in mice [47,48]. In 2019, Wang et al. found that **109D** (Figure 8) could attenuate lipopolysaccharide (LPS)-induced acute lung injury. A further mechanistic study indicated that **109D** reversed the LPS-induced increases of mRNA expression and protein levels of macrophage inflammatory protein-2 (MIP-2) and intercellular adhesion molecule-1 (ICAM-1) [49]. Compound **109D** also possessed neuroprotective activity by inhibiting the TLR4-mediated transforming growth factor-β-activated kinase-1(TAK1)/ nuclear factor kappa-B kinase 2 (IKK) /NF-κB, MAPKs, and Akt signaling pathways to exert anti-neuroinflammatory effects on LPS-activated microglia. In vivo experiments demonstrated that **109D** significantly inhibited microglial activation and proinflammatory factor expression in the mouse cortex and hippocampus after the LPS injection [50].

**Figure 10:**
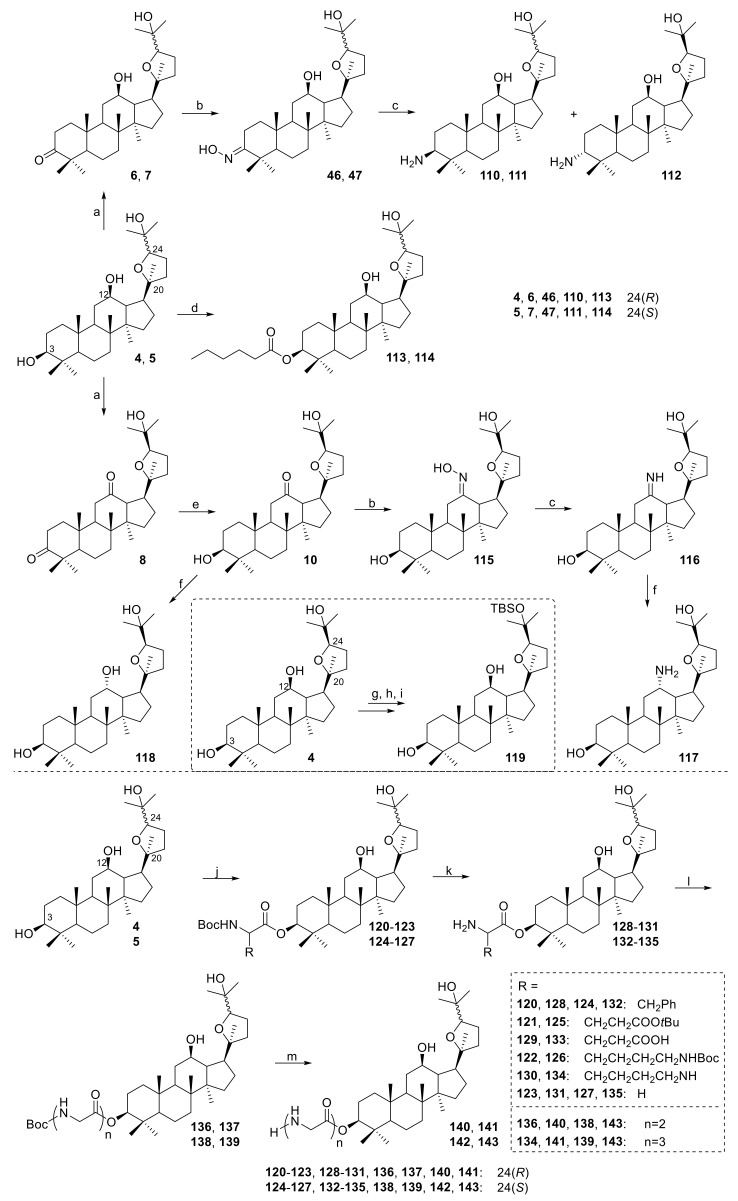
Synthesis of ocotillol-type derivatives **110**–**143**. Reagents and conditions: (a) PCC, CH_2_Cl_2_, r.t.; (b) Hydroxylamine hydrochloride, pyridine, 80 °C; (c) NaCNBH_3_, TiCl_3_, AcONH_4_, MeOH, r.t.; (d) *n*-Hexanoic acid, EDCI, DMAP, r.t.; (e) NaBH_4_, *i*-PrOH, r.t.; (f) NaBH_4_, MeOH, r.t.; (g) Ac_2_O, CH_2_Cl_2_, r.t.; (h) trifluoromethanesulfonic acid tert-butyldimethylsilyl ester (TBS-OTF), lutidine, r.t.; (i) KOH, MeOH, THF, r.t.; (j) Boc-amino acid, EDCI, DMAP, CH_2_Cl_2_, r.t.; (k) TFA, CH_2_Cl_2_, r.t.; (l) O-benzotriazole-N,N,N’,N’-tetraMethyl-uroniuM-hexafluorophosphate (HBTU), NEt_3_, DMF, r.t.; (m) TFA, CH_2_Cl_2_, r.t.

Ocotillol-type derivatives with NO-inhibitory activity were further studied (Figure 10) [51,52,53,54]. Derivatives **6**, **46**, **110**, **112**, **113**, **121**, **132** and **136** showed significant NO-inhibitory activities, while **115**, **116** and **119** had no obvious NO-inhibitory activities. Derivatives **46** and **136** exhibited the most potent NO-inhibitory activities and were even comparable to a steroid drug. Additionally, **46** and **136** significantly decreased LPS-induced TNF-α and IL-6 synthesis and iNOS and COX-2 expression via the NF-κB pathway.

**Figure 11:**
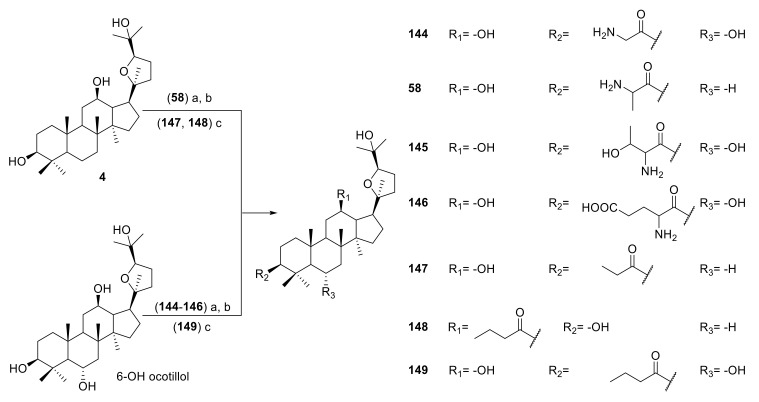
Synthesis of ocotillol-type derivatives **144**–**149**. Reagents and conditions: (a) Boc-amino acid, DMAP, EDCI, CH_2_Cl_2_, r.t.; (b) TFA, CH_2_Cl_2_, r.t.; (c) aliphatic acid, DCC, EDCI, CH_2_Cl_2_, r.t.

Wang et al. synthesized a series of ocotillol-type derivatives (Figure 11) [55,56,57]. Compound **144** had a protective effect on the lung function of experimental model mice with hormone-resistant asthma caused by non-typeable *Hemophilus influenzae* and improved their hormone resistance. Compounds **58** and **145**–**149** had inhibitory effects on the IL-6 expression and promoting effects on the IL-10 expression in the serum of rats induced by chronic obstructive pulmonary disease (COPD) caused by cigarette smoking.

**Figure 12:**
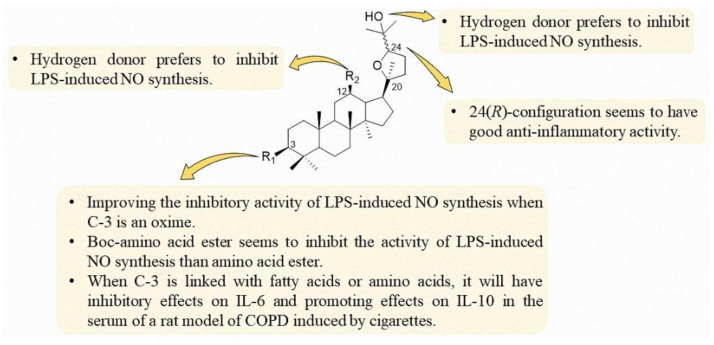
SAR of ocotillol-type derivatives with anti-inflammatory activity.

Based on the present research of ocotillol-type derivatives, a preliminary SAR of their anti-inflammatory effects is summarized, as shown in Figure 12. The 24(*R*)-configuration is preferred for the anti-inflammatory activity. An oxime at C-3 is preferred for good inhibitory activity of LPS-induced NO synthesis. Boc-amino groups seem to be preferred to inhibit the activity of LPS-induced NO synthesis than amino groups at C-3. A hydrogen donor at C-12 is preferred to inhibit LPS-induced NO synthesis. A fatty acid or amino acid group at C-3 has inhibitory effects on the expression of IL-6 and promotes the expression of IL-10 in the serum of a rat model of COPD induced by cigarettes.

### 3.3. Anticancer Effects of Ocotillol-Type Derivatives

**Figure 13:**
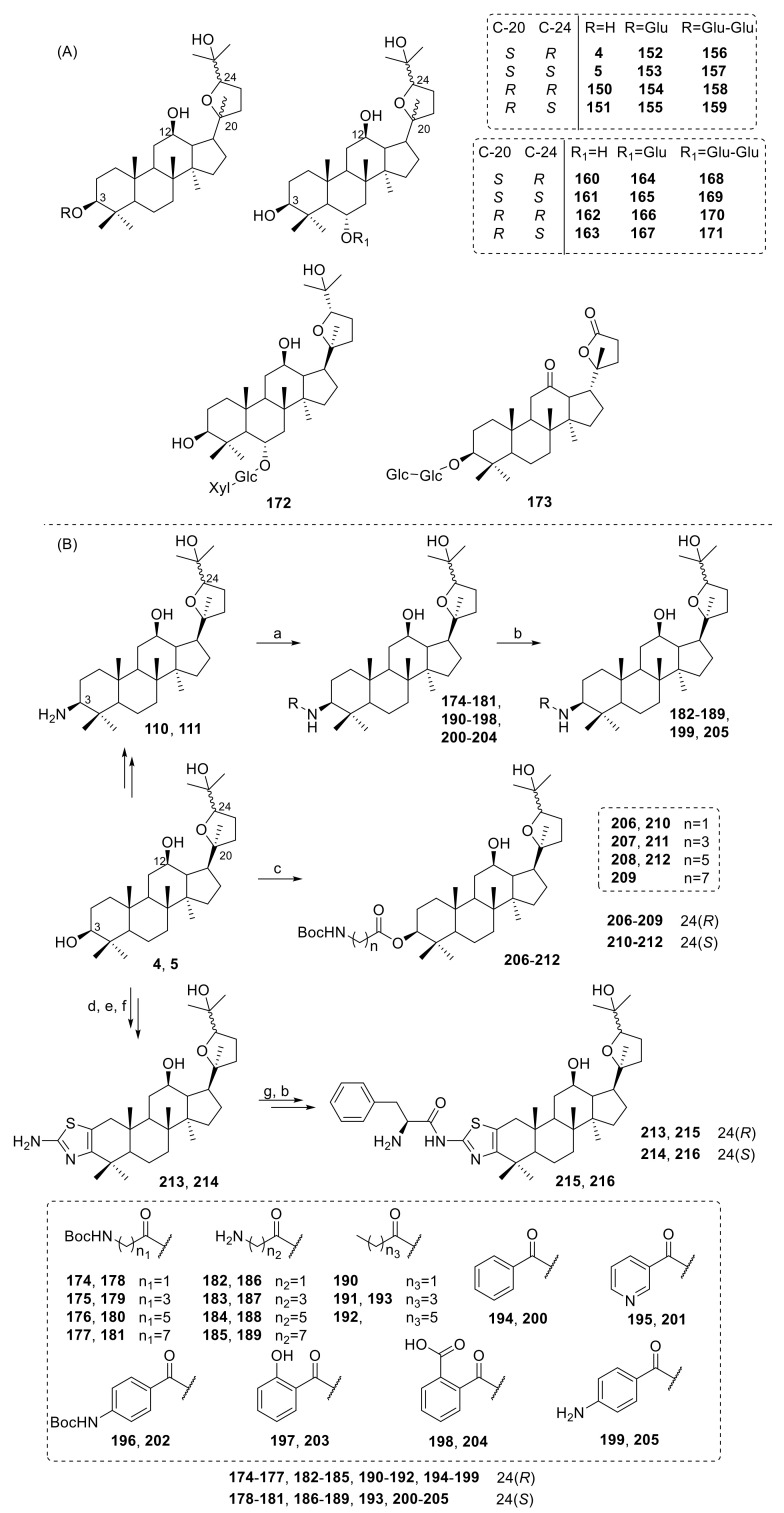
(**A**) Structures of ocotillol-type derivatives **150**–**173**. (**B**) Synthesis of ocotillol-type derivatives **174**–**216**. Ac = acetyl; Glc = β-d-glucopyranosyl; Xyl = β-d-xylopyranosyl; reagents and conditions: (a) ROH, HBTU, NEt_3_, DMF, r.t.; (b) TFA, CH_2_Cl_2_, r.t.; (c) DMAP, EDCI, CH_2_Cl_2_, r.t.; (d) anhydrous CH_2_Cl_2_, PCC, r.t.; (e) pyridinium tribromide, CH_2_Cl_2_, r.t.; (f) thiourea, CH_3_OH, ref.; (g) Boc-phenyl alanine, DMAP, EDCI, CH_2_Cl_2_, r.t.

The antitumor effect of ocotillol-type derivatives is mainly concentrated in the ocotillol monomer or in substances directly extracted from plants; thus, there are few reports on its structural modification [58,59]. **172** (Figure 13A) showed effective antitumor-promoting activity on a mouse hepatic tumor and mouse skin [60,61]. A series of ocotillol-type derivatives had been studied for their cytotoxic activity against HeLa, A549 and MCF-7 cancer cells. Pharmacological experiments on HeLa cells showed that ocotillol-type derivatives had cytotoxicity. Among them, compounds **5**, **152** and **173** (Figure 13A) possessed good activities with IC_50_ values of 11.53 ± 0.49 μM, 4.58 ± 0.66 μM and 19.84 ± 1.10 μM toward HeLa cells, respectively [62]. Compounds **162**, **163**, **167** and **166** showed reduced cell viabilities toward HeLa cells at 48.59%, 47.39%, 52.82% and 59.02% at 100 μg/mL, respectively [63].

Pharmacological results indicated that ocotillol-type derivatives had anticancer potential, and the configurations at C-20 or C-24 and the number of glycosyl units at C-3 could have an important influence on the cytotoxicity in vitro. There are only a small number of studies on ocotillol-type derivatives with anticancer activity, and thus, there is an opportunity to increase the number of ocotillol-type derivatives with anticancer activity.

### 3.4. Reversal of Multidrug Resistance in Cancer by Ocotillol-Type Derivatives

Ocotillol enhanced doxorubicin-induced cell death in p53 wild-type cancer cells [64]. Additionally, doxorubicin has a strong anticancer effect, but dose-dependent cardiotoxicity limits its clinical applications. Ocotillol-type ginseng reduced plasma creatine kinase and creatine kinase-MB isoenzyme levels and helped to reduce cardiotoxicity [65,66,67,68].

Wang et al. proved that ocotillol-type ginsenosides were substrates of P-glycoprotein (P-gp), and the pharmacological effects of ocotillol were the result of decreased efflux of digoxin across Caco-2 cell monolayers. In vivo experiments on mice showed that the inhibition of the 24(*R*)-epimer on P-gp was stronger than its counterpart [69]. This suggested that ocotillol-type ginseng may be a new type of drug resistance reversal agent.

**Figure 14:**
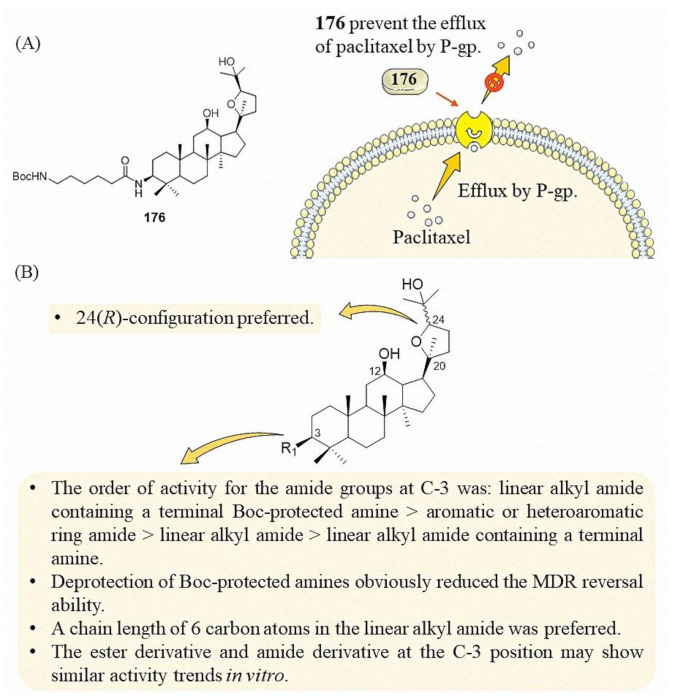
(**A**) Mechanism proposed for the reversal of multidrug resistance in cancer by ocotillol-type derivatives. (**B**) SAR of ocotillol-type derivatives with drug resistance reversal properties in cancer.

Pharmacological experiments showed that **110** significantly reversed the resistance of ABCB1-overexpressing SW620/Ad300 and HEK/ABCB1 cells to paclitaxel and vincristine (Figure 13B). A further mechanistic study showed that **110** reversed ABCB1-mediated MDR by competitively inhibiting the drug efflux function of ABCB1 [70]. On this basis, Ren et al. synthesized a series of derivatives (Figure 13B). Compounds **175**–**177**, **185**, **192**, **194** and **203** have demonstrated a promising capability to reverse drug resistance, with compound **176** showing slight superiority [71,72]. Importantly, a xenograft model of KBV200 cells in nude mice showed that oral **176** significantly enhanced the inhibitory effect of paclitaxel on tumor growth. The inhibition of paclitaxel in vivo is 17.9%, while the inhibition of paclitaxel with **176** is 53.75%. In vitro, mechanistic studies suggested that **176** could inhibit P-gp-mediated rhodamine123 efflux function via stimulation of P-gp-ATPase activity (Figure 14A). This indicated that ocotillol-type amide derivatives were substrates of P-gp, and it also showed that ocotillol-type amide derivatives were excellent drug resistance reversal agents.

Ocotillol ester derivatives with Boc-amino groups also have drug resistance reversal activity (Figure 13B) [73]. Compared with the positive drug verapamil, compounds **206**–**212** showed good paclitaxel enhancing effect on KBv200 cells at a concentration of 10 μM. Generally speaking, compare with amide derivatives, ester derivatives are prone to hydrolysis in vivo; therefore, ester derivatives may not have drug resistance reversal activity in vivo. Compared with compounds **174**–**180** and **206**–**212**, amide bond and ester bond have no effect on its activity in vitro, and almost all of these compounds with Boc-amino showed good drug resistance reversal activity, suggested that ocotillol-type derivatives containing Boc-amino group should be further enriched. Moreover, Bi et al. synthesized ring-A fused aminothiazole derivatives of ocotillol, compounds **215** and **216** possessed a remarkable multidrug resistance reversal activity higher than verapamil (Figure 13B). SAR of ring-A fused aminothiazole derivatives needs further research [74].

Based on the present research of ocotillol-type derivatives, a preliminary SAR of their multidrug resistance reversal ability in cancer cells is summarized in Figure 14B. The 24(*R*)-configuration is preferred for the reversal of multidrug resistance in cancer. A linear alkyl amide containing a terminal Boc-protected amine at C-3 shows the best drug resistance reversal activity. Aromatic or heteroaromatic ring amide is better than linear alkyl amide or linear alkyl amide containing a terminal amine. Deprotection of Boc-protected amines obviously reduced the MDR reversal ability. A length of six carbon atoms in the alkyl chain of the linear alkyl amide is preferred, whether the *N*-terminus is Boc-protected or not. The ester derivatives and amide derivatives at the C-3 position may show similar activity trends in vitro.

### 3.5. Nervous System Effects of Ocotillol-Type Derivatives

Ginseng is a traditional herb and has been widely used for the treatment of neurological disorders [75]. In 2013, the protective effect of **109D** on a rat model of Parkinson’s disease was studied. This research showed that **109D** had anti-Parkinson activity by inhibiting free radical formation and stimulating endogenous antioxidant release. Pretreatment based on oral administration of **109D** significantly improved the motor balance, coordination and apomorphine-induced rotations in 6-OHDA-lesioned rats [76,77].

Compound **109D** had inhibitory effects on the cognitive function of Tg-APPswe/PS1dE9 mice by inhibiting the expression of amyloid β-protein and amyloid-β-peptide (1–40) in the cortex and hippocampus, restoring the activities of superoxide dismutase and glutathione peroxidase, and decreasing the production of malondialdehyde in the cortex [78]. Compound **109D** showed a protective effect against mild cognitive impairment (MCI-like pathological changes) by reducing the accumulation of advanced glycation end products and expression of the receptor of advanced glycation end-products [79]. Compound **109D** attenuated memory disorders in the Morris water maze by promoting the transport of amyloid beta A4 and amyloid precursor protein from the cytoplasm to the plasma membrane and reducing the abnormally high expression of β-site APP cleaving enzyme 1 in the hippocampus and cortex of SAMP8 mice [80].

Compound **109D** may also be a candidate for stroke treatment. Compound **109D** inhibited the over-activation of μ-calpain and reduced the calcium calmodulin kinase II-α, reduced the degradation of sarcoplasmic/endoplasmic reticulum ATPase-2, and alleviated endoplasmic reticulum stress in transient middle cerebral artery occlusion rats [81]. Additionally, **109D** also accelerated the oxygen- and glucose deprivation-induced promotion of microglial myelin debris phagocytosis and reinforced the RhoA-ROCK signaling pathway through the regulation of complement receptor 3 [81,82]. Neutrophils and macrophages are promising targets for the treatment of cerebral ischemia. Compound **109D** inhibited the induction of neutrophils and macrophages to N1 and M1 phenotypes and promoted the polarization of neutrophils and macrophages to N2 and M2 phenotypes [83].

**Figure 15:**
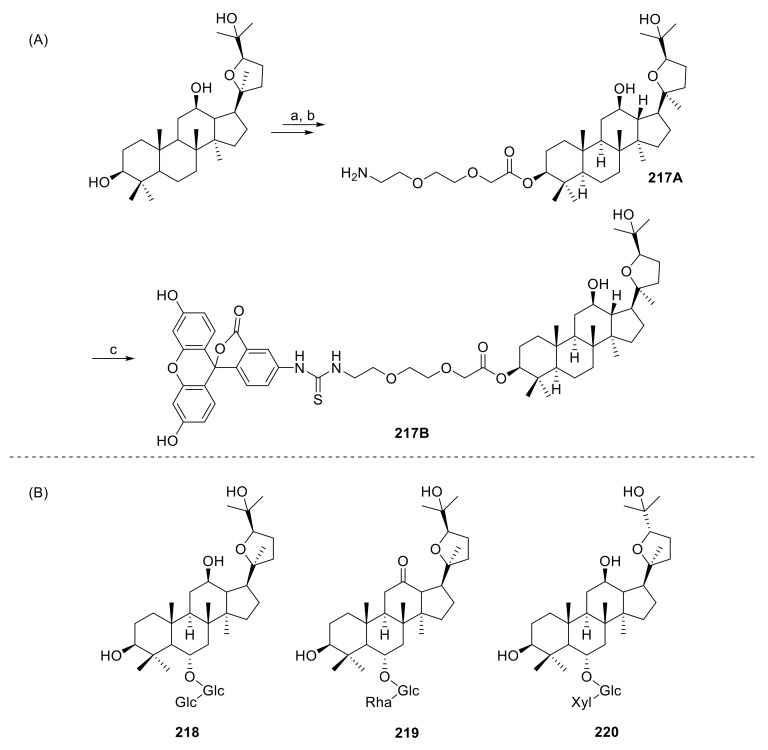
(**A**) Synthesis of ocotillol-type probe **217B**. (**B**) Structures of **218**–**220**. Glc = β-d-glucopyranosyl; Xyl = β-d-xylopyranosyl; Rha = α-l-rhamnopyranosyl. Reagents and conditions: (a) 2-[2-(Fmoc-amino)ethoxy]ethoxy acetic acid, EDCI, DMAP, CH_2_Cl_2_, r.t.; (b) DEA, CH_2_Cl_2_, r.t.; (c) FITC, Et_3_N, DMF, r.t.

Quyen et al. evaluated the antidepressant-like activity of **109C** and **218** by a tail suspension test and a forced swimming test in mice (Figure 15B). The results showed that the stress model caused an increase of MDA and a decrease of glutathione levels in the mouse brain. This proved that **109C** and **218** had antidepressant effects [84].

### 3.6. Effects of Ocotillol-Type Derivatives on the Cardiovascular System

Ocotillol-type derivatives protect from myocardial ischemic injury by reducing the area of the myocardial ischemia and the levels of necrosis and lactate dehydrogenase in the serum to enhance the anti-free-radical actions of heart tissues [85,86,87]. Bi et al. found that when **4** and its epimer **5** were tested in cultured myocardiocytes with anoxia/re-oxygen injury, only **4** had protective effects [88]. In 2017, Yang et al. synthesized an ocotillol-type small-molecule fluorescent probe **217B** with anti-myocardial ischemia-reperfusion injury activity (Figure 15A). This tool may help to understand the mechanism of how ocotillol-type derivatives protect against myocardial ischemia [89].

Oral administration of compound **4** ameliorated aconitine-induced arrhythmias [90]. Compound **4** reduced the incidence of arrhythmia in mice and shortened the duration time of ventricular tachycardia. Further research proved that oral administration of compound **4** prolonged action potential duration, reduced action potential amplitude in ventricular myocytes, reduced L-type calcium peak current in a dose-dependent manner, and inhibited delayed rectifier K^+^ channels, but not inward rectifier K^+^ channels.

### 3.7. Other Pharmacological Activities of Ocotillol-Type Derivatives

Ocotillol-type ginsenoside **219** (Figure 15B), discovered from the stems and leaves of *Panax quinquefolium* L., increased the production of superoxide dismutase and glutathione, decreased malondialdehyde production, and increased the expression level of nuclear correlation factor 2 and heme oxygenase-1 in A549 cells. These results showed that compound **219** significantly inhibited hydrogen peroxide-induced oxidative stress and had a protective effect on the oxidative damage of lung epithelial cells [91].

Ocotillol-type ginsenosides have anti-melanogenic activity as **220** showed a good melanogenesis effect with an IC_50_ value of 37 μM, but the mechanism of its anti-melanogenic effect is still not clear (Figure 15B) [92]. Additionally, ocotillol may also have a protective effect against gastric ulcers. Ocotillol increased the expression of NO, superoxide dismutase, epidermal growth factor and the epidermal growth factor receptor, and decrease the expression of endotelin-1 and nitric oxide synthase, which is a similar effect as omeprazole [93]. In addition, ocotillol also has antiviral activity, and it could enhance the neuronal activity of mice [24,94,95].

## 4. Conclusions and Future Perspectives

In this review, the main chemical modifications of ocotillol-type derivatives and the SARs for their antibacterial, anti-inflammatory and reversal of multidrug resistance in cancer were summarized. In the past few years, ocotillol has attracted considerable interest in the medicinal chemistry society owing to its promising multiple pharmacological activities, especially antibacterial activity. Nevertheless, ocotillol-type derivatives exhibit limited water solubility, low systemic exposure, slow clearance and imprecise mechanism of action. Toxicity has greatly hindered its clinical applications [96,97,98,99,100,101,102]. To advance ocotillol-type derivatives into clinical therapies, there remain to be several issues and new directions for future research in the area.

(1) Rational design of new ocotillol-type derivatives with increased water solubility, good ADME. For example, through polyethylene glycol modification or preparation techniques such as micronization, solid dispersion, self-microemulsion, inclusion techniques, etc., to improve water solubility. Formulation design of sustained- or controlled-release system should be used to maintain an effective blood concentration and decrease side effects.
(2) Ocotillol, an active ingredient in ginseng, has already been proved to have multiple pharmacological activities; however, its precise molecular targets that responsible for the potent biological activity are currently not well understood. Therefore, it is important to further design and synthesize a new ocotillol-type probe to explore possible mechanisms and identify the molecular target.
(3) Currently, there is still much chemical space to be explored. The main chemical modifications performed to date have focused on the hydroxyl groups on ring A, while the skeleton structures and ring C modifications have been limited.
(4) As many of the current studies are limited to in vitro studies, whether ocotillol is effective in vivo must be validated in the future.
(5) Combination drugs have various significant advantages, including production additive or synergistic effects, reducing side effects, treatment failure rates and slow down the development of drug resistance [103]. The development of ocotillol-based combination drugs would be a useful strategy. For example, the combination of ocotillol with other antibacterial drugs to reduce treatment failure rates.

## Abbreviations

Structure–activity relationship (SAR); 20(*S*)-protopanaxadiol (20(*S*)-PPD); prostaglandin E_2_ (PGE_2_); acetyl (Ac); benzyl (Bn); β-d-glucopyranosyl (Glc); Bbnzoyl (Bz); *Staphylococcus aureus* (*S. aureus*); *Bacillus subtilis* 168 (*B. subtilis*); community associated methicillin-resistant *S. aureus* (MRSA USA300); minimum inhibitory concentration (MIC); 1-ethyl-3(3-dimethylpropylamine) carbodiimide (EDCI); 4-dimethylaminopyridine (DMAP); fractional inhibitory concentration index (FICI); *Escherichia coli* (*E. coli*); *Pseudomonas aeruginosa* (*P. aeruginosa*); *Acinetobacter baumannii* (*A. baumannii*); minimum bactericidal concentration (MBC); nitric oxide (NO); tumor necrosis factor (TNF-α); interleukin-6 (IL-6); interleukin-10 (IL-10); interleukin-17 (IL-17); interleukin-23 (IL-23); interferon-γ (IFN-γ); cyclooxygenase-2 (COX-2); lipopolysaccharide (LPS); macrophage inflammatory protein-2 (MIP-2); chronic obstructive pulmonary disease (COPD); Intercellular adhesion molecule-1 (ICAM-1); transforming growth factor-β-activated kinase-1(TAK1); nuclear factor kappa-B kinase 2 (IKK); Toll-like receptor 4 (TLR4); nuclear factor-kappa B (NF-κB); **Mitogen-activated protein kinase** (MAPK); protein kinase B (Akt); Inducible NOS (iNOS); P-glycoprotein (P-gp); β-d-xylopyranosyl (Xyl); α-l-rhamnopyranosyl (Rha); trifluoromethanesulfonic acid tert-butyldimethylsilyl ester (TBS-OTF); O-benzotriazole-N,N,N’,N’-tetraMethyl-uroniuM-hexafluorophosphate (HBTU); trifluoroacetic acid (TFA); pyridinium chlorochromate (PCC); Oak Ridge Thermal Ellipsoid Plot Program for Crystal Structure Illustrations (ORTEP).
